# Impact of near continuous low dose rate neutron irradiation on pregnancy outcomes in mice

**DOI:** 10.1038/s41526-024-00438-9

**Published:** 2024-12-19

**Authors:** Jon G. Steller, Rebecca S. Blue, April E. Ronca, Andrew Goodspeed, Theresa L. Powell, Thomas Jansson

**Affiliations:** 1 University of Colorado Anschutz Medical Campus, Department of Obstetrics & Gynecology, Division of Maternal Fetal Medicine, Aurora, CO USA; 2 University of California, Irvine, Department of Obstetrics & Gynecology, Irvine, CA USA; 3 University of Texas Medical Branch, School of Public and Population Health, Galveston, TX USA; 4 NASA Ames Research Center, Space Biosciences Division, Mountain View, CA USA; 5 Wake Forest School of Medicine, Department of Obstetrics & Gynecology, Winston-Salem, NC USA; 6 University of Colorado Anschutz Medical Campus, University of Colorado Cancer Center, Aurora, CO USA; 7 University of Colorado Anschutz Medical Campus, Department of Pharmacology, Aurora, CO USA; 8 University of Colorado Anschutz Medical Campus, Department of Obstetrics & Gynecology, Division of Reproductive Sciences, Aurora, CO USA; 9 University of Colorado Anschutz Medical Campus, Department of Pediatrics, Section of Neonatology, Aurora, CO USA

**Keywords:** Outcomes research, Developmental biology

## Abstract

The effects of galactic cosmic radiation on reproductive physiology remain largely unknown. We determined the impact of near-continuous low-dose-rate Californium-252 neutron irradiation (1 mGy/day) as a space-relevant analog on litter size and number of resorptions at embryonic day (E) 12.5 (*n* = 19 radiated dams, *n* = 20 controls) and litter size, number of resorptions, fetal growth, and placental signaling and transcriptome (RNA sequencing) at E18.5 (*n* = 21 radiated dams, *n* = 20 controls) in pregnant mice. A significantly increased early resorption rate and decreased placental weight were observed in irradiated mice. There were no statistically significant differences in litter size, fetal weight, length, or malformation rate between the groups. Near-continuous radiation had no significant effects on the mechanistic target of rapamycin (mTOR), endoplasmic reticulum stress or inflammatory signaling, rate of double-stranded DNA breaks, and had minimal effects on gene expression in the placenta. These data suggest that near-continuous, low-level galactic cosmic radiation has a limited impact on pregnancy outcomes.

## Introduction

As NASA and the commercial spaceflight industry prepare for long-duration exploration spaceflight missions to the Moon and Mars, the possibility of extra-terrestrial colonization remains a remote, but conceivable, future. Independent of future colonization goals, gynecological and reproductive considerations remain important to astronaut health and mission risk mitigation^1–4^. Specifically, understanding the impact that space radiation may have on fertility, fecundity, pregnancy, and fetal development is important for the protection of current crewmembers’ reproductive health. In the future, such knowledge will be simultaneously critical for both risk mitigation and human reproductive success during long-duration spaceflight or colonization^3–5,6^.

Among other sustained physiological challenges in the space environment, space radiation poses a potential risk to pregnancy and fetal viability. Studies determining the effect of spaceflight-related stressors on fertility, placentation, embryogenesis and development, and maternal health are lacking^3,4,7^. Extrapolating conclusions from terrestrial human and animal studies exploring the risks of radiation during pregnancy are severely limited given differences in types of radiation, total doses, dose-rates, and time of exposures that deviate substantially from those that would be encountered in spaceflight^8–12^. The current understanding of the risks associated with exposure to radiation in pregnancy is based upon a small body of information derived from relatively large, acute radiation exposures. Epidemiologic studies of pregnant women exposed to radiation, including atomic bomb survivors and women exposed to radiation for medical reasons (i.e., imaging or cancer treatment), have demonstrated elevated risk of miscarriage from acute gamma irradiation exposures of 50 mGy between 0-2 weeks of gestation^13–15^ (Table 1). The risk for fetal growth restriction and congenital anomalies begins at doses exceeding 200 mGy occurring during fetal organogenesis^13–15^. Further, exposures at 8-15 weeks of gestation of >60 mGy gamma irradiation may be associated with a higher incidence of fetal intellectual disability^13–15^. Additional studies have determined the impacts of terrestrial radiation exposures on pregnancy in rodents^16,17^ and reported that radiation can lead to fetal growth restriction^18–21^, a decrease in litter size^17^, a higher rate of resorption (miscarriage) or stillbirth^18^, and fetal anatomical anomalies^22–24^. However, even these studies were constrained by the use of large one-time or recurrent exposures of 10-300 mGy of x- or gamma irradiation, rather than low-dose, and low dose-rate continuous radiation exposures to heavy- or mixed-ion species more reflective of the space environment. Further, detailed analyses of pregnancy outcomes, including placental development and function are often omitted.

**Table 1:** Acute radiation exposure thresholds for increased risk of negative fetal impact in humans

Weeks Post-Conception	Estimated Dosing Thresholds
**0–2 weeks**	50-100 mGy – All-or-nothing resorption/miscarriage effect
**2–8 weeks**	> 200 mGy – Risk of induced developmental defects 200-250 mGy – Risk of fetal growth restriction
**8–15 weeks**	60-310 mGy – Risk of severe intellectual disability 200 mGy – Risk of microcephaly
**16–25 weeks**	250-280 mGy – Risk of severe intellectual disability

Adapted from the American College of Obstetrics & Gynecology’s Committee Opinion Number 723.

Radiation in the space environment primarily consists of constant, cumulative, and low dose-rate exposures to galactic cosmic radiation (GCR), consisting of complex, mixed species radiation^25–27^. Such exposures are strikingly different from the acute, one-time, or recurrent single-ion exposures used in previous studies of the impact of radiation on pregnancy. Most terrestrial radiation studies rely on x-ray or gamma irradiation exposures. In contrast, GCR includes protons and charged heavy ions that can deposit energy along their entire path, leading to more densely clustered collisions within tissues/cells and a higher rate of double-strand breaks in deoxyribonucleic acid (DNA)^10,11,25^. The International Commission on Radiological Protection (ICRP) has estimated relative biological effectiveness (RBE) for different types of radiation occurring in space including protons (RBE 2-5), neutrons (a continuous function ranging from 2.5-20 depending upon neutron energy), and heavy ion particles (RBE 20), respectively^26,27^. However, it is important to note that an appropriate model of the RBEs for spaceflight-associated irradiation specific to pregnancy-related effects has never been developed. Even so, differences in total dose exposures, dose-rates, radiation species (and their associated biological effects) limit the ability to extrapolate health outcomes from terrestrial studies to the space environment or to predict potential radiation-induced reproductive complications during spaceflight. Whether cumulative low-dose and dose-rate space radiation exposures during pregnancy result in similar biological sequelae to large, acute, or even recurrent exposures remains unclear.

On average, the estimated terrestrial natural background radiation dose-rate received by individuals at sea level is <0.003 mSv/day^28^. In comparison, measured daily exposures on the International Space Station (ISS) range from 0.2-0.4 mGy/day^29^. The estimated total body dose to astronauts traveling outside of low-Earth orbit (LEO) is approximately 1-2 mSv per day in interplanetary space and is estimated to be approximately 0.5-1 mSv/day on the surface of Mars^27,29–35^; recent advanced modeling of spaceflight radiation exposure to the female genitourinary system estimates doses of approximately 0.29 mGy-Eq/day to the uterus^36^. However, amniotic fluid will provide further fetal shielding for a developing fetus. Approximating these values from predicted dosing to blood-forming organs, the extrapolated daily dose-rates to a developing fetus in the uterus on Mars are estimated to range from 0.13-0.8 mGy-Eq/day^29,30,32–36^. Robust studies determining the impact of cumulative, low total dose, and low dose-rate exposures in these ranges on pregnancy are lacking.

While there is a paucity of research elucidating the effects of radiation on placental function, disruption from radiation-induced damage is known to impact fetal growth trajectory or body composition and predispose the human fetus to later disease^13–15^. Experiments in rodents and non-human primates have demonstrated that changes in placental function precede the occurrence of fetal growth restriction (FGR)^37,38^ strongly suggesting that inhibition of key placental signaling pathways is a cause of FGR rather than a secondary consequence of slowed fetal growth. Some key signal markers that have been found to be altered in the case of radiation-induced stress are presented in Table 2^16,22^.

**Table 2:** Previously reported differentially expressed genes in mouse placentas following radiation exposure^16,22^

Gene Symbol	Gene Name	Function
Afp	alpha fetoprotein	Fetal counterpart of serum albumin
Aqp1	aquaporin 1	Water channel protein
Brca1	BRCA1 DNA repair associated	Tumor suppressor
Cdkn1c	cyclin-dependent kinase inhibitor 1C	Negative regulator of cell proliferation
Egr1	early growth response 1	Transcriptional regulator
Ercc6	ERCC excision repair 6, chromatin remodeling factor	Transcription-coupled excision repair
Gem1	ERMES complex Ca(2+)-binding regulatory GTPase	Regulatory GTPase
Gpx1	glutathione peroxidase 1	Protects cells against oxidative damage
Hbb-y	hemoglobin Y, beta-like embryonic chain	Heme binding activity; Hemoglobin alpha binding activity; and oxygen carrier activity
Hif1a	hypoxia inducible factor 1 subunit alpha	Master regulator of cellular and systemic homeostatic response to hypoxia
Hist3h2a	histone cluster 3, H2a	Nuclear proteins that are responsible for the nucleosome structure
Hspa1b	heat shock protein family A (Hsp70) member 1B	Involved in ubiquitin-proteasome pathway
Igf2	insulin like growth factor 2	Involved in development and growth
Igfb2	insulin like growth factor binding protein 2	Binds insulin-like growth factors I and II
Krt19	keratin 19	Keratin responsible for the structural integrity of epithelial cells
Lama1	laminim subunit alpha 1	Major component of the basement membrane and implicated in cell adhesion, differentiation, migration, signaling, neurite outgrowth, and metastasis
Lcor	ligand dependent nuclear receptor corepressor	Transcriptional corepressor
Lepr	leptin receptor	Stimulates gene transcription via activation of cytosolic STAT proteins
Ler3	immediate early response 3	Regulation of response to DNA damage
Mlh1	mutL homolog 1	Part of the DNA mismatch repair system
Mmp2	matrix metallopeptidase 2	Type IV collagenase
Mos	MOS proto-oncogene, serine/threonine kinase	Activates the MAP kinase cascade
Ndrg1	N-myc downstream regulated 1	p53-mediated caspase activation and apoptosis
Parp1	poly(ADP-ribose) polymerase 1	Recovery of cell from DNA damage
Pcdh12	protocadherin 12	Cellular adhesion protein
Polb	DNA polymerase beta	Base excision and repair
Rad51	RAD51 recombinase	Homologous recombination and repair of DNA
Raf1	Raf-1 proto-oncogene, serine/threonine kinase	Activates the dual specificity protein kinases MEK1 and MEK2
Sod1	superoxide dismutase 1	Binds copper and zinc ions and is one of two isozymes responsible for destroying free superoxide radicals in the body
Timp2	TIMP metallopeptidase inhibitor 2	Maintenance of tissue homeostasis by suppressing the proliferation of quiescent tissues
Tnf	tumor necrosis factor	Involved in the regulation of cell proliferation, differentiation, apoptosis, lipid metabolism, and coagulation
Tpbpa	trophoblast specific protein alpha	Acts upstream of or within JNK cascade
Trp53	transformation related protein 53	Responds to diverse cellular stresses to regulate target genes that induce cell cycle arrest, apoptosis, senescence, DNA repair, or changes in metabolism
Ube2	ubiquitin like modifier activating enzyme 7	Retinoid target that triggers promyelocytic leukemia (PML)/retinoic acid receptor alpha (RARalpha) degradation and apoptosis
Vldlr	very low density lipoprotein receptor	Important roles in VLDL-triglyceride metabolism and the reelin signaling pathway
Xpa	DNA damage and repair factor	Zinc finger protein plays a central role in nucleotide excision repair

In this study, we determined the impact of analog radiation exposure to chronic background GCR on litter size, fetal growth, and placentation in pregnant mice. We tested the hypothesis that exposure of pregnant mice throughout gestation to near-continuous low total dose and dose-rate neutron irradiation (1 mGy/day) results in an increased resorption rate, fetal growth restriction, and decreased maternal weight gain, without a significant increase in congenital malformation rate. To explore the mechanistic link between radiation exposure and pregnancy outcomes, we further hypothesized that radiation exposure leads to inhibition of the placental mechanistic target of rapamycin (mTOR) signaling^37–40^, activation of endoplasmic reticulum (ER) stress^41–43^ and inflammatory pathways^44–46^, and an increase the frequency of double-stranded DNA breaks^16^. Signaling pathways associated with unfolded protein response, cellular stress, inflammatory cytokine response, and apoptosis were selected for analysis. We additionally employed an unbiased discovery approach (RNAseq) to identify additional placental pathways altered by near-continuous low-dose-rate neutron irradiation.

## Results

Phenotypical outcomes in irradiated and control dams at embryonic day 12.5 (E12.5) and E18.5 are presented in Table 3. At both gestational ages, litter sizes were similar in the two groups. At both E12.5 and 18.5, an increased number of early resorptions was observed in the irradiated animals; this difference was significant at E18.5 but did not reach the level of statistical significance at E12.5. Exposure to continuous low-dose-rate neutron irradiation also decreased maternal weight gain at E12.5 (–16.0%, *p* < 0.023) and E18.5 (–10.9%, *p* < 0.062), though the maternal weight gain difference was statistically significant only at E12.5. Placental weights were decreased (–12.1%, *p* < 0.013) in the radiation group at E18.5. There were no significant differences in fetal weight or length. Two anomalies (fetal hydrops and exencephaly) were observed among the 147 fetuses in the E18.5 radiation group; however, the study was not powered to detect a difference in congenital malformation rate.

**Table 3:** Phenotypic characteristics of control and Irradiated litters at E12.5 and E18.5

E12.5	Control (*n* = 20) (Mean +/– SEM)	Radiation (*n* = 19) (Mean +/– SEM)	*P*-value
**Implantation Rate^**	8.15+/– 0.49	8.47+/– 0.45	0.461
**Total Early Resorptions**~	11	20	**0.092**
**Viable Litter Size^**	7.60+/– 0.50	7.42+/– 0.56	0.847
**Maternal Weight Gain**^**#**^ **(g)**	6.58+/- 0.27	5.53+/– 0.33	0.023*

E18.5	Control (*n* = 20)(Mean +/– SEM)	Radiation (*n* = 20)(Mean +/– SEM)	*P*-value
**Implantation Rate^**	8.20+/– 0.43	8.60+/– 0.47	0.353
**Total Resorptions**~	6	18	0.011*
**Viable Litter Size^**	7.90+/– 0.42	7.60+/– 0.50	0.550
Fetal Weight* (g)	1.16+/– 0.01	1.19+/– 0.02	0.311
**Fetal Length**^**#**^ **(mm)**	23.50+/– 0.11	23.32+/– 0.18	0.395
**Fetal Anomalies**^**&**^	0	2	n/a
**Placental Weight^ (g)**	0.091+/– 0.00	0.080+/– 0.00	0.013*
**Maternal Weight Gain**^**#**^ **(g)**	17.76+/– 0.59	15.82+/– 0.60	0.062

Statistical analysis: ^#^Unpaired T-tests, ~Fischer’s Exact Test, or ^Mann-Whitney were used following a D’Agostino-Pearson normality test. ^&^The study was not powered to evaluate anomalies. Significant differences are identified by asterisks.

Significant *p*-values are bolded.

**Fig. 1:**
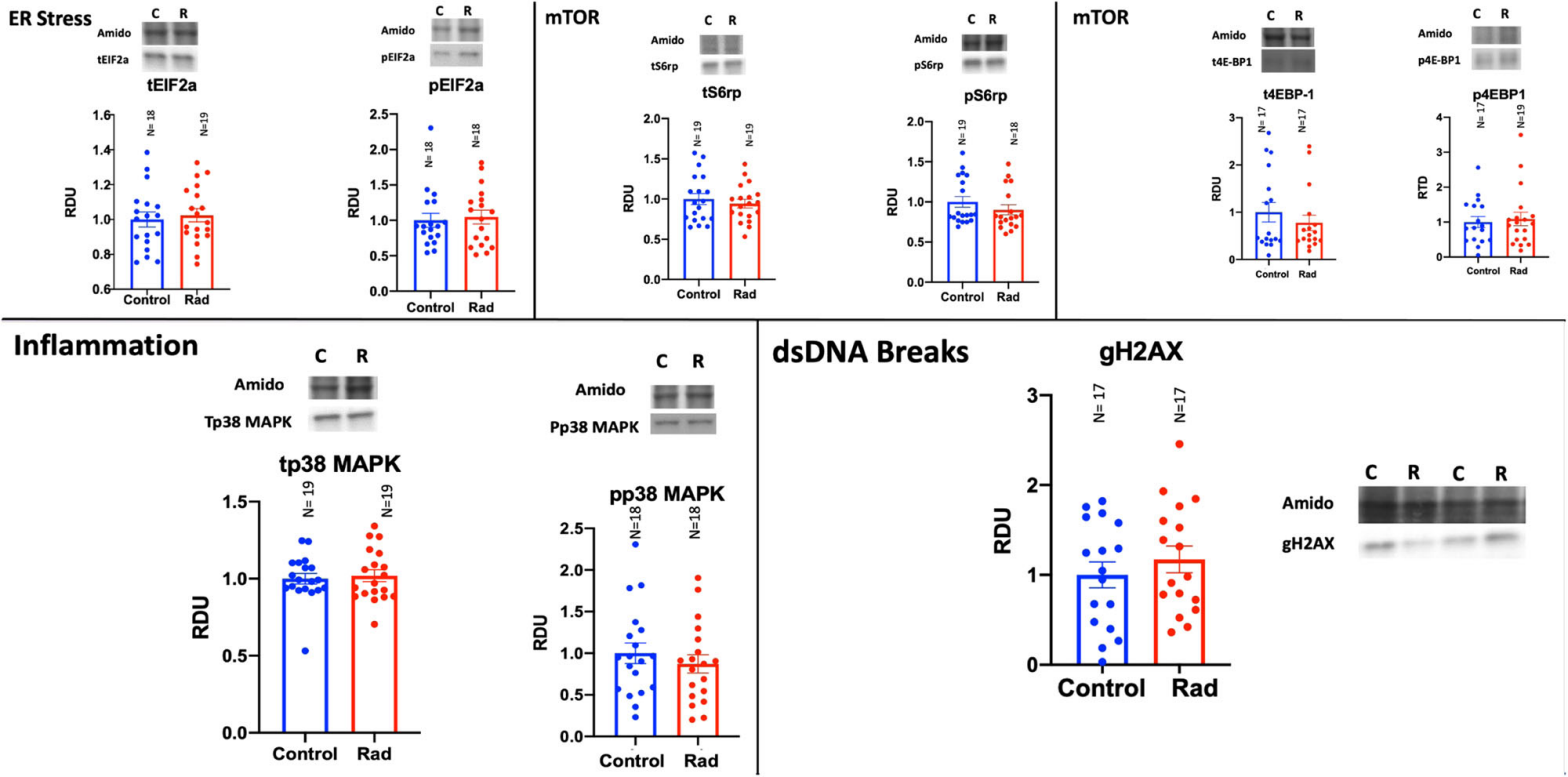
Representative samples of the placental signaling Western blots performed. No differences in signaling pathways for mTOR (tS6rp/pS6rp, t4EBP1/p4EBP1), ER stress (tEIF2a/pEIF2a), inflammation (tp38 MAPK/pp38 MAPK), and double-stranded DNA breaks (gH2AX). Full Western Blots for Fig. 1 are supplied in Supplementary Information.

Table 4 and Fig. 1 present the outcomes of the placental signaling data. There were no significant differences in the activity of placental mTOR (tS6rp/pS6rp, t4EBP1/p4EBP1), ER stress (tEIF2a/pEIF2a) or inflammation (tp38 MAPK/pp38 MAPK) signaling pathways. Similarly, we found no evidence of an increase in double-stranded DNA breaks (gH2AX) in placentas of the radiation group.

**Table 4:** Total expression and phosphorylation of proteins in key placental signaling pathways

Targeted Protein *(N control/N rad)*	Control *Mean +/- SEM*	Radiation *Mean +/- SEM*	*P*-value
tEIF2a (18/19)*	1.00 ± 0.059	1.01 ± 0.057	0.951
pEIF2a~ (18/18)	1.00 ± 0.098	1.046 ± 0.097	0.817
tp38 MAPK~ (19/19)	1.00 ± 0.035	1.021 ± 0.039	0.773
pp38 MAPK* (18/18)	1.00 ± 0.123	0.871 ± 0.110	0.442
tS6rp* (19/19)	1.00 ± 0.068	0.943 ± 0.053	0.511
pS6rp* (19/18)	1.00 ± 0.066	0.900 ± 0.061	0.281
t4EBP1~ (17/17)	1.00 ± 0.207	0.777 ± 0.162	0.792
p4EBP1~ (17/19)	1.00 ± 0.157	1.088 ± 0.192	0.925
gH2AX* (17/17)	1.00 ± 0.144	1.173 ± 0.148	0.410

For each protein target, the control sample band mean density was assigned an arbitrary value of 1. Data are presented relative to control. The relative density of the target protein in each lane was divided by the density of the corresponding total protein band as a loading control and then groups were compared using unpaired t tests (*) or Mann-Whitney U tests (~) following a D'Agostino Pearson Normality Test.

### Placental transcriptome

**Fig. 2:**
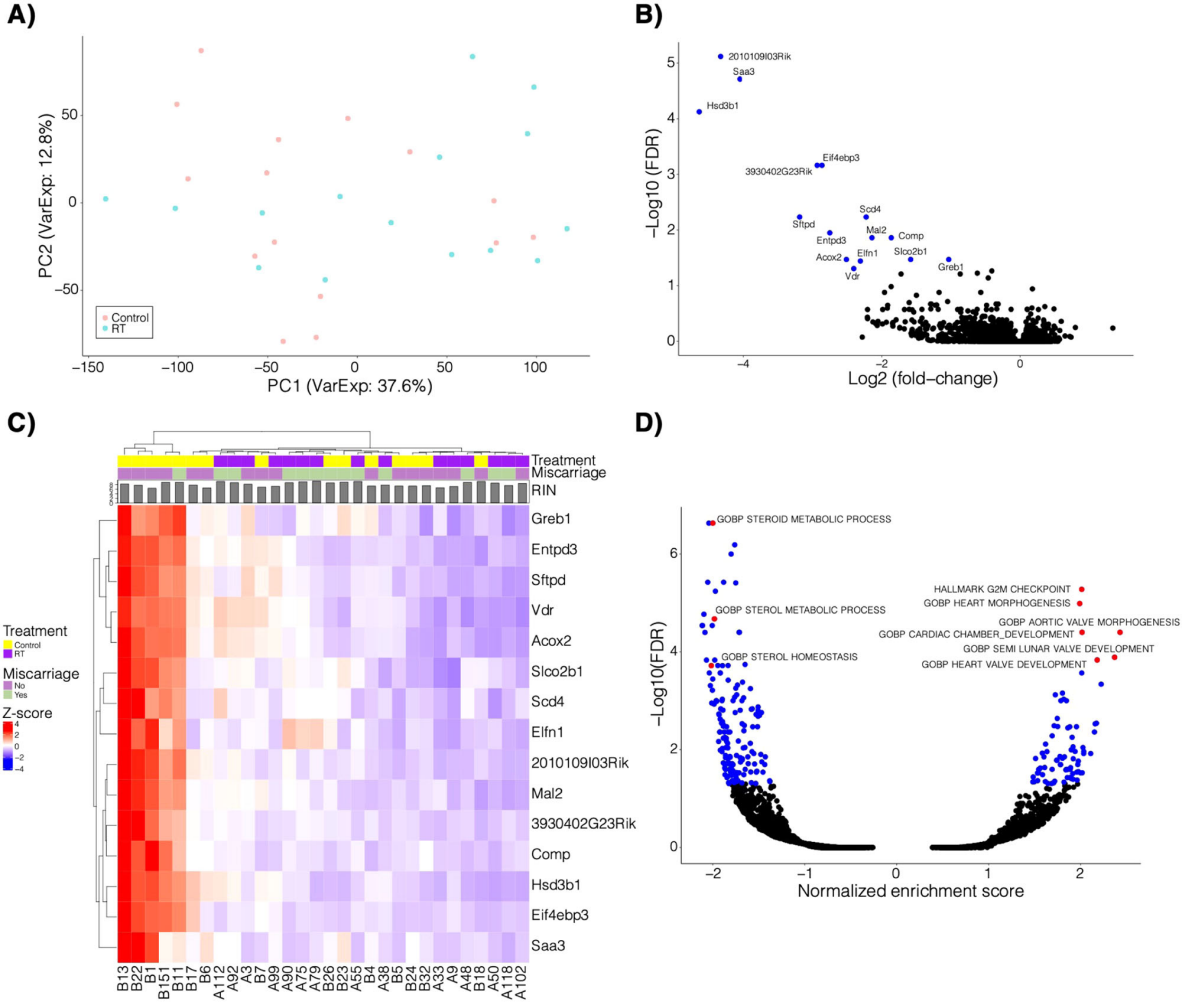
Placental transcriptomics. **A** Principal component analysis of the RNA sequencing data with sample groups indicated (RT=radiation-exposed placenta). **B** Volcano plot of the fold-change and -log10 FDR of the differential expression comparing radiation to control. A negative fold change indicates enrichment in the control group. **C** Heatmap of the z-score normalized expression of the 15 significant genes highlighted from **B**. Treatment, resorption, and RIN (RNA integrity number) is also shown. RIN is a standard algorithm for assigning integrity values to RNA measurements. **D** Gene set enrichment analysis of fold-change from the comparison in B. Select gene sets are highlighted and labeled. A negative normalized enrichment score indicates enrichment in the control group.

RNA sequencing was performed on placentas of radiated and control dams. Principal component analysis (PCA) revealed high intragroup variability in the data (Fig. 2A). Only 15 differentially expressed genes (false discovery rate (FDR) < 0.05 [5%]) were identified. All were downregulated in the radiation treated mice (Fig. 2B, C, Supp Table 1). Gene set enrichment analysis (GSEA) was performed on the fold-change from this comparison because of the lack of significant genes (Fig. 2D). There were 241 significant gene sets; the top 25 most significant gene sets are presented in Supplementary Table 2. Several gene sets related to steroid metabolism and homeostasis were downregulated in the radiation group. Three genes (Hsd3b1, Acox2, Vdr) involved in steroid metabolism were significantly downregulated. Several of the gene sets that were upregulated in the radiation group involve cardiac development and morphogenesis, and another significant gene set governed cellular proliferation.

## Discussion

This is the first study determining the effects of near-continuous low dose-rate neutron irradiation, a spaceflight GCR analog, on pregnancy outcomes. Significant findings included decreased maternal weight gain in early gestation, increased rate of early resorptions, and a decreased placental weight in irradiated mice. Despite these statistically significant findings, the biological significance of these findings is uncertain because litter sizes were not affected by radiation, the activity of key placental signaling pathways was not changed and fetal growth restriction was not observed. Moreover, near-continuous low dose-rate neutron irradiation did not increase the frequency of DNA double-strand breaks in the placenta and we found very limited changes in the placental transcriptome. Overall, this study suggests that low-level radiation from GCR may have only a limited impact on pregnancy outcomes.

Irradiation was associated with decreased maternal weight gain, but only at E12.5; weights of irradiated dams were not significantly different from controls at E18.5. Though an increased rate of early resorptions was observed in irradiated animals, litter sizes were not significantly different between irradiated and control animals, and thus differences in the numbers of live pups do not explain the lower maternal weight gain observed in the first part of pregnancy in the radiation group. Although food and water consumption were not directly measured, it is plausible that radiation exposure led to decreased maternal appetite and/or water intake^46,47^. Dam irradiation was additionally associated with decreased placental weight. However, fetal growth was unaffected, suggesting some degree of compensation in placental efficiency in response to low dose-rate neutron irradiation. None of the placental signaling pathways under study were adversely affected by radiation, consistent with maintained placental function despite near-continuous irradiation.

While the study was not powered to determine differences in fetal malformation rate, it is notable that the two severe abnormalities observed, exencephaly and hydrops fetalis, both occurred in the radiation group. While sporadic congenital malformation rates vary from 0.1–10% in murine strains, most of these anomalies are less severe (for example, eye defects, polydactyly, and otocephaly)^48^. As hydrops can be observed in association to central nervous system anomalies like exencephaly, the two anomalies noted in our study may be related findings. Exencephaly has been previously reported following exposure to much higher, acute radiation (150 mGy x-irradiation) in early pregnancy in mice (E1.5)^49^; however, hydrops has not been explicitly associated with fetal irradiation in the literature. Given the significantly increased early resorption rates, fetal malformations may occur early in gestation and result in resorption, in general agreement with prior evidence that developing embryos are most sensitive to radiation in early gestation. These observations are also in line with human studies in which early gestation radiation exposure is associated with the highest radiation sensitivity (see Table 1)^13–15^.

We performed exploratory RNA sequencing to identify potential changes in placental gene expression in response to low dose-rate neutron irradiation. Ultimately there were very few differentially expressed genes found. Further, upon review of the candidate genes from previous radiation studies of the placenta^16,22^, expression of the genes outlined in Table 2 was not found to be significantly altered following radiation exposure in our study. These findings suggest important differences between dose and dose-rates of exposures and resultant clinical sequelae, and that high-dose and high dose-rate terrestrial exposure outcomes may not correlate to potential GCR exposure outcomes in spaceflight. Similarly, the frequency of double-strand DNA breaks can be identified via surrogate analysis of gH2AX, a protein involved in the recruitment and localization of DNA repair proteins^50,51^. Here, our study results indicated no significant difference in the expression of gH2AX between control and irradiated placentas, suggesting that the low total dose and dose-rate exposure in this experiment were insufficient to lead to a significant increase in DNA double-stranded breaks or associated recruitment of repair proteins.

There are several experimental design limitations to this study, including the low cumulative dose received by mice due to their short gestation compared to higher-order mammals. Maternal weight was measured, but detailed data collection regarding food and water intake was lacking, restricting our ability to fully evaluate potential contributory factors to decreased maternal weight gain in early gestation. The sample size was not powered to identify the effects of radiation on congenital malformation rate, and RNA sequencing data was limited by the evaluation of only one placenta per litter in a subset of litters. Dams were euthanized at E18.5 for timely collection of placentas and, although major effects of radiation on gestational length is unlikely, risks of preterm delivery or delivery complications could not be studied directly. While this study utilized radiation exposure at a space-relevant dose-rate and ion species, exposures lacked the complexity of the mixed-species GCR environment. Finally, just as extrapolation of radiation dose and dose-rate to the actual space environment is theoretical and complex, extrapolation of results from mice models to human clinical outcomes must be done with caution given significant interspecies differences.

Despite these limitations, this study provides important data regarding the effects of low-dose and dose-rate ion exposure during pregnancy, laying a critical foundation for future studies. The sum of our findings is that while a GCR-analog exposure can lead to increased resorptions in mice pregnancy, it is overall reassuring that a space-relevant exposure did not appear to affect fetal growth and viability in a mammalian model that shares a similar hemochorial placenta to humans.

## Methods

### Animal Model

All protocols were approved by the Institutional Animal Care and Use Committee at Colorado State University (CSU). Eight-week-old C57BL/6 mice (The Jackson Laboratory, Bar Harbor, ME, USA) were group-housed and received ad libitum standard diet (Teklad 2018) and water. Mice were harem mated at 9–15 weeks of age and the presence of a vaginal plug the morning after mating was defined as embryonic day (E) 0.5. After confirming copulatory plugs, 80 pregnant mice were randomly allocated to control or radiation groups for the duration of pregnancy (E0.5-E18.5) at NASA’s CSU Neutron Radiation Facility^52,53^. Radiation groups were exposed to near-continuous neutron irradiation (21 hours/day) using Californium-252 at a dose-rate of 1 mGy/day as determined by dosimeters located in the mouse cages. Californium-252 emits 2.34 × 10^12^ neutrons per second per gram by spontaneous fission; this species has been utilized as a terrestrial GCR analog for the simulation of high linear energy transfer (LET) exposures expected during spaceflight^52^^,53^. For the dose from neutrons as a function of LET, the dose-averaged value was calculated to be 68 keV/µm. The mixed fluence is a combination of neutrons and photons directly from the source and spallation. This approach resulted in an average daily dose to each animal of 1mGy per day. Control groups were maintained in a vivarium approximately 100 yards away on the same campus and were exposed only to the background radiation rate of 0.005 mGy/day which is typical for the elevation at CSU. Food and water consumption could not be strictly determined due to time limitations of staff operating within the radiation facility; however, pregnancy weight trajectories were monitored. All animals were maintained on a 12-hour light/dark cycle with light hours from 0700-1900 daily.

### Animal sample processing

On gestational day 12.5 (E12.5; mid-pregnancy), one exposure group (19 dams) and one control group (20 dams) were weighed and then euthanized by carbon dioxide inhalation followed by bilateral thoracotomy and laparotomy for tissue collection. Blood was collected via cardiac puncture, with subsequent centrifugation and serum collection. Serum samples were flash-frozen in liquid nitrogen and stored in a –80 °C freezer for subsequent analysis. Hysterectomy was performed, and rates of early resorption were recorded. Each successful pregnancy implantation site was opened to remove the fetus. The uterus (and the placenta in situ) were dissected out and placed in zinc salts fixation solution at 4 °C for 24 hours, dehydrated through a series of graded ethanol baths to displace water, and infiltrated with paraffin wax.

On gestational day 18.5 (E18.5; full term, with anticipated pregnancy duration of ~19.5 days), the remaining radiation exposure group (21 dams) and control group (20 dams) were euthanized in an identical fashion for tissue collection including maternal serum and uteri. One dam in the radiation group was excluded due to the delivery of her pups just prior to euthanasia leaving 20 for analysis. Following hysterectomy, rates of early or late resorption were recorded via gross inspection. Fetuses were removed and placed in iced phosphate buffered saline (PBS) before body weight and length were recorded and pups were evaluated for anomalies. Late resorptions were defined by fetal remains with no discernable anatomy. Placentas were removed, the amnion was removed, and placentas were weighed, flash-frozen in liquid nitrogen, and stored at –80 °C until subsequent analyses, including placental cell signaling and gene expression studies.

### Western blot analysis

Frozen placental samples from the E18.5 groups were homogenized, and Western blot analysis was used to determine the activity of mTOR, endoplasmic reticulum (ER) stress, and inflammatory signaling pathways as well as the presence of DNA double-stranded breaks. Activation of mTOR was assessed by phosphorylation of ribosomal protein S6 (rpS6), and eukaryotic translation initiation factor 4E-binding protein 1 (4E-BP1). Additional analysis included total and phosphorylated p38-MAPK expression to assess inflammatory pathways^44,45,54^, total and phosphorylated eIF2α expression as a functional readout of ER stress^41–43^, and gH2AX expression to assess dsDNA breaks^50,51^.

Commercial antibodies were obtained from Cell Signaling Technology, Boston, MA, USA. 10–15 mg of total protein were loaded onto a sodium dodecyl sulfate-polyacrylamide gel electrophoresis (SDS-PAGE), and proteins were separated at a constant 80 V for 15 minutes followed by 100 V for and additional 45–60 mins. Proteins were transferred onto polyvinylidene difluoride (PVDF) or nitrocellulose membranes, and transferred overnight (~18 hours) at a constant voltage of 35 V. After transfer, membranes were stained with Amido black (Sigma-Aldrich, St. Louis, MO) for 45 seconds and a G:Box (Syngene, Frederick, MD) was used to determine the intensity of the total protein signal, which was subsequently used to normalize the relative abundance of the target proteins. Membranes were de-stained, washed and blocked in tris-buffered saline (TBS) (wt/vol) plus 0.1% Tween® 20 (vol/vol) with 5% nonfat dry milk (Bio-Rad, Hercules, CA) or 5% bovine serum albumin for 1 h at room temperature, per manufacturer protocol. Membranes were subsequently incubated with primary antibodies overnight at 4 °C then washed and incubated with the appropriate secondary peroxidase-labeled antibodies for 1 h. Bands were visualized using enhanced chemiluminescence detection reagents (GE Healthcare, Chalfont, St. Giles, Buckinghamshire, UK). GeneGnome XRQ, a chemiluminescence CCD-based system, was used to capture images of the Western blots in the G:Box; GeneSys software (Genesys, Menlo Park, CA) was used to perform densitometry and compare chemiluminescent Western blots to the Amido black normalization images. For each protein target, the mean density of the control sample bands was assigned an arbitrary value of 1, and data are presented relative to control.

### RNA Sequencing

RNA sequencing was performed on one randomly selected placenta from each of 15 irradiated E18.5 litters and 15 control litters. The RNA from each of the 30 individual placentas was isolated using a Qiagen RNeasy mini kit following the manufacturer’s instructions. Next, a reverse transcriptase reaction was performed using the Qiagen Quantitect RT kit according to manufacturer’s instructions (Qiagen, Hilden, Germany). The resulting cDNA was then subjected to quantitative PCR. RNA purity, quantity, and integrity was determined with NanoDrop (ThermoFisher Scientific, Waltham, MA) and TapeStation 4200 (Agilent, Santa Clara, CA) analysis prior to RNA-seq library preparation. The Universal Plus mRNA-Seq library preparation kit with NuQuant was used (Tecan, Männedorf, Switzerland) with an input of 200 ng of total RNA to generate RNA-Seq libraries. Paired-end sequencing reads of 150 bp were generated on NovaSeq 6000 (Illumina, San Diego, CA) sequencer and de-multiplexed using bcl2fastq.

Illumina adapters and the first 12 base pairs of each read were trimmed using BBDuk (BBMAP; www.sourceforge.net/projects/bbmap) and reads <50 bp post-trimming were discarded. Reads were aligned and quantified using STAR (2.6.0a)^55^ against the Ensembl mouse transcriptome (mg38.p6 genome release 96). Ensembl IDs were mapped to gene names and counts of genes with multiple IDs were aggregated. Low expression genes were removed if mean raw count <1 or mean counts per million (CPM) < 1 for the dataset. Reads were normalized to CPM using the edgeR R package (Bioconductor; Boston, MA)^56^. Differential expression was calculated using the *voom* function in the *limma* R package with RNA integrity numbers as a covariate^57^. Gene set enrichment analysis (GSEA) was performed using the full ranked list of genes by fold change for the indicated comparison and the fgsea R package^58^ using Hallmark, Gene Ontology Biological Processes, and KEGG gene sets from the Molecular Signatures Database^59,60^. A heatmap was generated with the ComplexHeatmap R package^61^ following z-score transformation. Additional plots were generated using the ggplot2 R package^62^.

### Statistical analysis

Comparisons were made between the radiation and control groups at each gestational age. Each litter was considered to be a single replicate given that each fetus is a non-independent observation sharing a common dam, genetic substrate, and host of environmental variables^63^. Following D’Agostino-Pearson normality tests, unpaired T-tests, Fischer’s Exact tests, or Mann-Whitney tests were used to compare phenotypic data between radiation and control groups. For analysis of placental signaling pathways, the mean density of the control sample bands was assigned an arbitrary value of 1, and data for each protein target were presented relative to the control. The relative density of the target protein in each lane was divided by the density of the corresponding total protein band as a loading control and then target protein abundance was compared between groups using unpaired T-tests.

## Supplementary information

Supplementary Information

## Data Availability

The data that support the findings of this study are available upon request form the corresponding author [JS]. Raw and processed RNA sequencing data are available in the Gene Expression Omnibus (GSE271237).
