# Supplementary material for: Impact of near continuous low dose rate neutron irradiation on pregnancy outcomes in mice
Source: NPJ Microgravity. 2024 Dec 19;10:113. doi: 10.1038/s41526-024-00438-9 (PMC11659551; doi:10.1038/s41526-024-00438-9)
Supplement: Supplementary file 1 — Supplementary Information [file 41526_2024_438_MOESM1_ESM.pdf]

Supplemental Table 1. Differentially expressed placental genes in response to irradiation.

Gene	Ave Expr	logFC	lfcSE	P Value	Adj P Value
2010109I03Rik	8.7631817	-4.327273	0.69692875	5.33E-10	7.61E-06
Saa3	6.97776764	-4.047831	0.68050677	2.71E-09	1.93E-05
Hsd3b1	9.60261727	-4.6364079	0.82018564	1.58E-08	7.51E-05
Eif4ebp3	5.67080987	-2.8633729	0.55328594	2.28E-07	0.000688838
3930402G23Rik	5.44045189	-2.9329896	0.56793712	2.41E-07	0.000688838
Sftpd	8.37245862	-3.1861324	0.67695276	2.52E-06	0.0058462
Scd4	6.28160701	-2.2247382	0.47536446	2.87E-06	0.0058462
Entpd3	7.92276366	-2.749815	0.6089283	6.31E-06	0.011250127
Comp	6.12500283	-1.8618017	0.42067056	9.61E-06	0.013777209
Mal2	6.13672579	-2.1408389	0.48383093	9.65E-06	0.013777209
Acox2	8.01869775	-2.5103933	0.59971462	2.84E-05	0.033889504
Slco2b1	8.32351041	-1.5823257	0.37809672	2.85E-05	0.033889504
Greb1	7.50739808	-1.0322687	0.24772904	3.09E-05	0.033889504
Elfn1	5.63669841	-2.3083295	0.55825049	3.55E-05	0.036190273
Vdr	9.13361522	-2.403874	0.59404333	5.20E-05	0.049431395

Table 1. Fifteen differentially expressed genes (all significantly downregulated at FDR < 0.05) were identified in placentas the radiation treated mice.

Supplemental Table 2. The top 25 most significant gene sets using fGSEA.

fGSEA GENE SET	Set Size	Enrich Score	NES	P Value	Adj P Value	Q Values	Rank
GOBP_NEGATIVE_REGULATION_OF_PEPTIDASE_ACTIVITY	158	-0.745256	-2.043041	1.00E-10	2.35E-07	2.07E-07	698
GOBP_STEROID_METABOLIC_PROCESS	218	-0.724378	-1.999553	1.00E-10	2.35E-07	2.07E-07	1251
GOBP_ORGANIC_HYDROXY_COMPOUND_METABOLIC_PROCESS	384	-0.633095	-1.761591	4.16E-10	6.51E-07	5.74E-07	1653
GOBP_NEGATIVE_REGULATION_OF_HYDROLASE_ACTIVITY	308	-0.649478	-1.800768	8.55E-10	1.00E-06	8.85E-07	698
HALLMARK_COAGULATION	109	-0.768328	-2.054814	4.69E-09	3.78E-06	3.34E-06	1201
GOBP_NEGATIVE_REGULATION_OF_PROTEOLYSIS	227	-0.681214	-1.881993	4.84E-09	3.78E-06	3.34E-06	1564
GOBP_REGULATION_OF_PEPTIDASE_ACTIVITY	319	-0.631132	-1.750248	5.83E-09	3.91E-06	3.44E-06	698
HALLMARK_G2M_CHECKPOINT	181	0.465857	2.0121441	9.01E-09	5.29E-06	4.66E-06	2057
GOBP_STEROID_BIOSYNTHETIC_PROCESS	135	-0.728668	-1.971851	1.11E-08	5.78E-06	5.09E-06	1003
GOBP_HEART_MORPHOGENESIS	185	0.4654061	1.9902064	2.20E-08	1.03E-05	9.11E-06	2851
GOBP_REGULATION_OF_PLASMA_LIPOPROTEIN_PARTICLE_LEVELS	71	-0.811176	-2.096713	4.00E-08	1.71E-05	1.51E-05	436
GOBP_STEROL_METABOLIC_PROCESS	127	-0.733661	-1.982160	5.43E-08	2.13E-05	1.87E-05	1003
GOBP_HUMORAL_IMMUNE_RESPONSE	112	-0.749493	-2.005858	8.23E-08	2.91E-05	2.56E-05	1087
KEGG_COMPLEMENT_AND_COAGULATION_CASCADES	44	-0.863037	-2.111329	8.95E-08	2.91E-05	2.56E-05	1215
GOBP_PROTEIN_LIPID_COMPLEX_SUBUNIT_ORGANIZATION	42	-0.869046	-2.116218	9.29E-08	2.91E-05	2.56E-05	436
GOBP_SMALL_MOLECULE_CATABOLIC_PROCESS	315	-0.618372	-1.714092	1.36E-07	4.00E-05	3.53E-05	1640
GOBP_CARDIAC_CHAMBER_DEVELOPMENT	130	0.492017	2.0161425	1.51E-07	4.00E-05	3.53E-05	2313
GOBP_AORTIC_VALVE_MORPHOGENESIS	28	0.7745539	2.4290479	1.60E-07	4.00E-05	3.53E-05	1834
GOBP_POST_TRANSLATIONAL_PROTEIN_MODIFICATION	315	-0.616939	-1.71012	1.62E-07	4.00E-05	3.53E-05	517
GOBP_PROTEIN_CONTAINING_COMPLEX_REMODELING	26	-0.913301	-2.085026	1.71E-07	4.02E-05	3.54E-05	436
GOBP_SEMI_LUNAR_VALVE_DEVELOPMENT	38	0.7061996	2.3698387	5.80E-07	0.000130	0.000114	2170
GOBP_HEART_VALVE_DEVELOPMENT	56	0.6145876	2.1803350	6.89E-07	0.000147	0.000130	2283
GOBP_CHOLESTEROL_EFFLUX	35	-0.864946	-2.068669	7.54E-07	0.000148	0.000130	263
GOBP_NEUTRAL_LIPID_METABOLIC_PROCESS	89	-0.746564	-1.978627	7.56E-07	0.000148	0.000130	1251
GOBP_LIPID_LOCALIZATION	340	-0.592956	-1.648712	9.68E-07	0.000182	0.000160	1328

Supplementary Figure 1

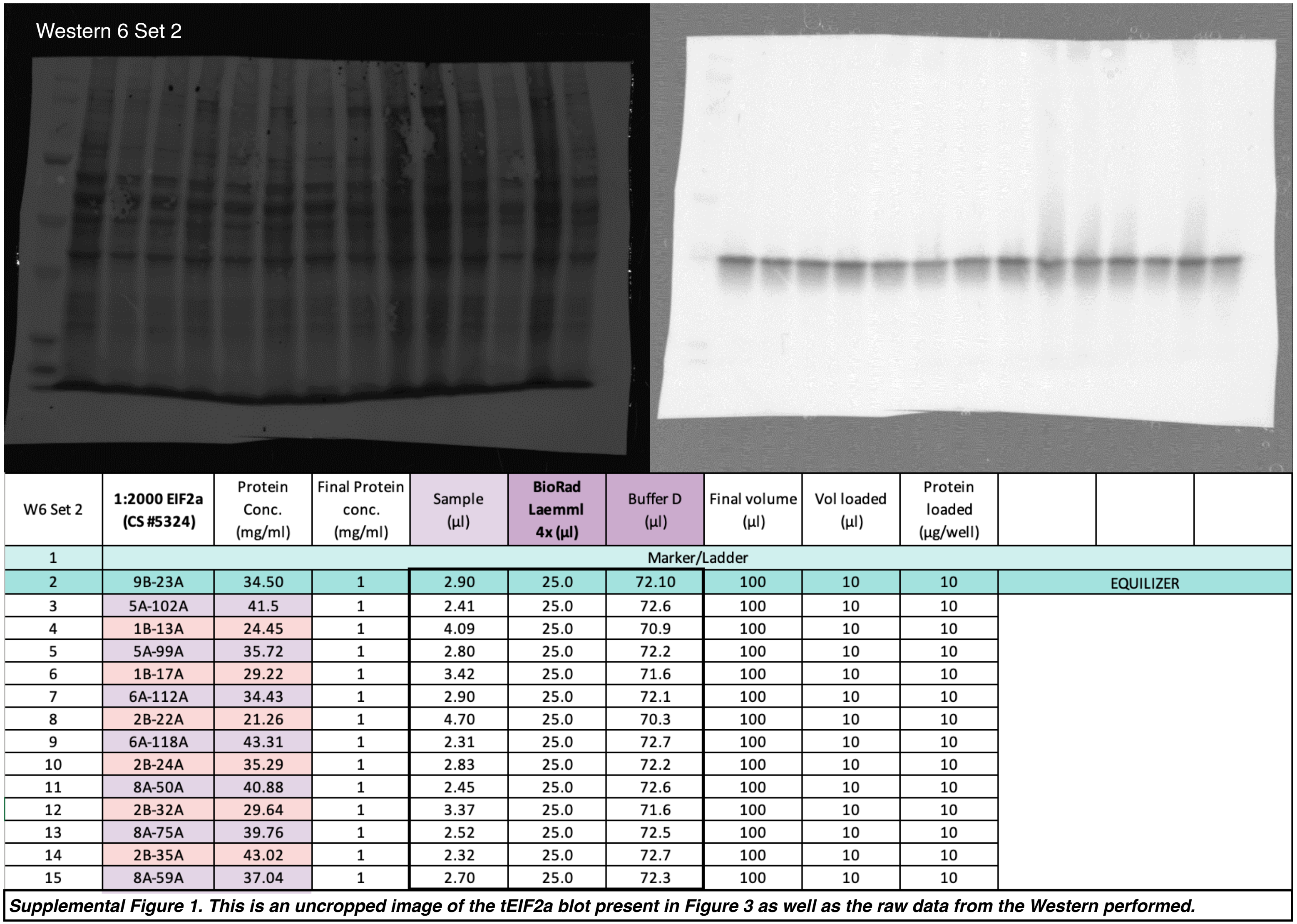

W6 Set 2	**1:2000 EIF2a (CS #5324)**	Protein Conc. (mg/ml)	Final Protein conc. (mg/ml)	Sample (µl)	**BioRad Laemml 4x (µl)**	Buffer D (µl)	Final volume (µl)	Vol loaded (µl)	Protein loaded (µg/well)			
1	Marker/Ladder											
2	9B-23A	34.50	1	2.90	25.0	72.10	100	10	10	EQUILIZER		
3	5A-102A	41.5	1	2.41	25.0	72.6	100	10	10			
4	1B-13A	24.45	1	4.09	25.0	70.9	100	10	10			
5	5A-99A	35.72	1	2.80	25.0	72.2	100	10	10			
6	1B-17A	29.22	1	3.42	25.0	71.6	100	10	10			
7	6A-112A	34.43	1	2.90	25.0	72.1	100	10	10			
8	2B-22A	21.26	1	4.70	25.0	70.3	100	10	10			
9	6A-118A	43.31	1	2.31	25.0	72.7	100	10	10			
10	2B-24A	35.29	1	2.83	25.0	72.2	100	10	10			
11	8A-50A	40.88	1	2.45	25.0	72.6	100	10	10			
12	2B-32A	29.64	1	3.37	25.0	71.6	100	10	10			
13	8A-75A	39.76	1	2.52	25.0	72.5	100	10	10			
14	2B-35A	43.02	1	2.32	25.0	72.7	100	10	10			
15	8A-59A	37.04	1	2.70	25.0	72.3	100	10	10			

Supplemental Figure 1. This is an uncropped image of the tEIF2a blot present in Figure 3 as well as the raw data from the Western performed.

Supplementary Figure 2

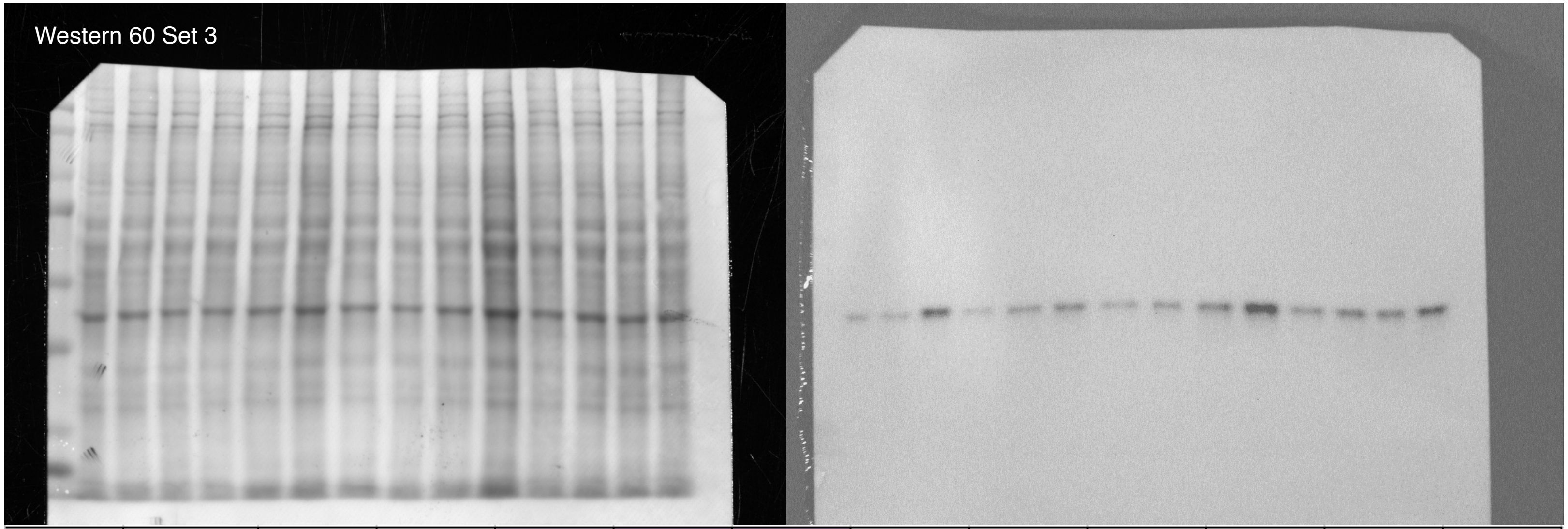

W60 Set 3	**1:1000 pEIF2a (#3398)**	Protein Conc. (mg/ml)	Final Protein conc. (mg/ml)	Sample (µl)	**BioRad Laemml 4x (µl)**	Buffer D (µl)	Final volume (µl)	Vol loaded (µl)	Protein loaded (µg/well)			
1	Marker/Ladder											
2	9B-23A	34.50	1	2.03	17.5	50.47	70	10	10	EQUILIZER		
3	8A-47A	43.06	1	0.58	6.3	18.2	25	10	10			
4	7B-18A	42.99	1	0.58	6.3	18.2	25	10	10			
5	8A-55A	34.47	1	0.73	6.3	18.0	25	10	10			
6	7B-39A	38.09	1	0.66	6.3	18.1	25	10	10			
7	8A-49A	33.59	1	0.74	6.3	18.0	25	10	10			
8	9B-26A	43.1	1	0.58	6.3	18.2	25	10	10			
9	8A-90A	35.29	1	0.71	6.3	18.0	25	10	10			
10	9B-34A	42.64	1	0.59	6.3	18.2	25	10	10			
11	10A-79A	30.59	1	0.82	6.3	17.9	25	10	10			
12	9B-11A	38.42	1	0.65	6.3	18.1	25	10	10			
13	8A-59A	37.04	1	0.67	6.3	18.1	25	10	10			
14	12B-151A	40.55	1	0.62	6.3	18.1	25	10	10			
15	5A-96A	33.20	1	2.11	17.5	50.4	70	10	10			

Supplemental Figure 2. This is an uncropped image of the pEIF2a blot present in Figure 3 as well as the raw data from the Western performed.

Supplementary Figure 3

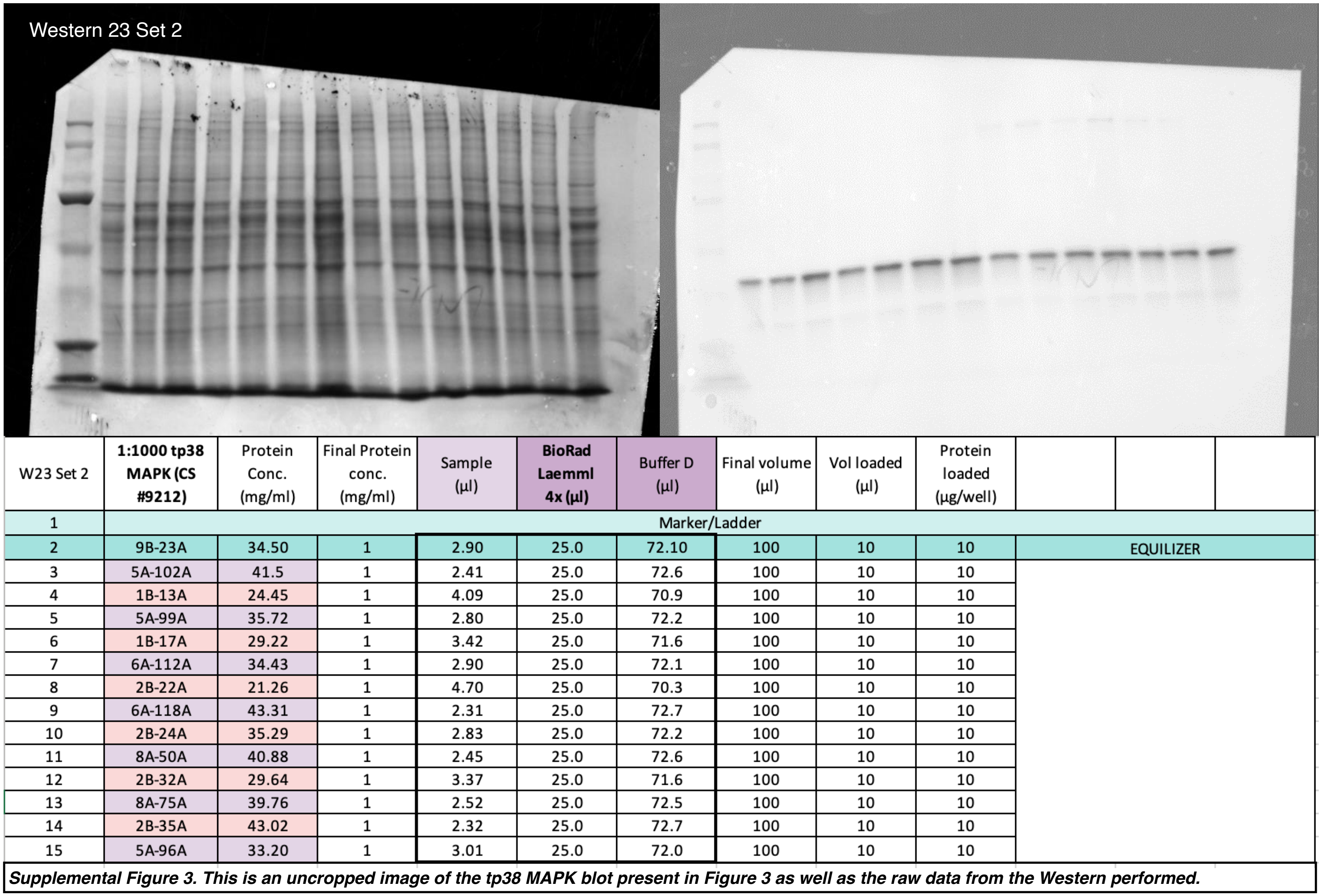

W23 Set 2	**1:1000 tp38 MAPK (CS #9212)**	Protein Conc. (mg/ml)	Final Protein conc. (mg/ml)	Sample (µl)	**BioRad Laemml 4x (µl)**	Buffer D (µl)	Final volume (µl)	Vol loaded (µl)	Protein loaded (µg/well)			
1	Marker/Ladder											
2	9B-23A	34.50	1	2.90	25.0	72.10	100	10	10	EQUILIZER		
3	5A-102A	41.5	1	2.41	25.0	72.6	100	10	10			
4	1B-13A	24.45	1	4.09	25.0	70.9	100	10	10			
5	5A-99A	35.72	1	2.80	25.0	72.2	100	10	10			
6	1B-17A	29.22	1	3.42	25.0	71.6	100	10	10			
7	6A-112A	34.43	1	2.90	25.0	72.1	100	10	10			
8	2B-22A	21.26	1	4.70	25.0	70.3	100	10	10			
9	6A-118A	43.31	1	2.31	25.0	72.7	100	10	10			
10	2B-24A	35.29	1	2.83	25.0	72.2	100	10	10			
11	8A-50A	40.88	1	2.45	25.0	72.6	100	10	10			
12	2B-32A	29.64	1	3.37	25.0	71.6	100	10	10			
13	8A-75A	39.76	1	2.52	25.0	72.5	100	10	10			
14	2B-35A	43.02	1	2.32	25.0	72.7	100	10	10			
15	5A-96A	33.20	1	3.01	25.0	72.0	100	10	10			

Supplemental Figure 3. This is an uncropped image of the tp38 MAPK blot present in Figure 3 as well as the raw data from the Western performed.

Supplementary Figure 4

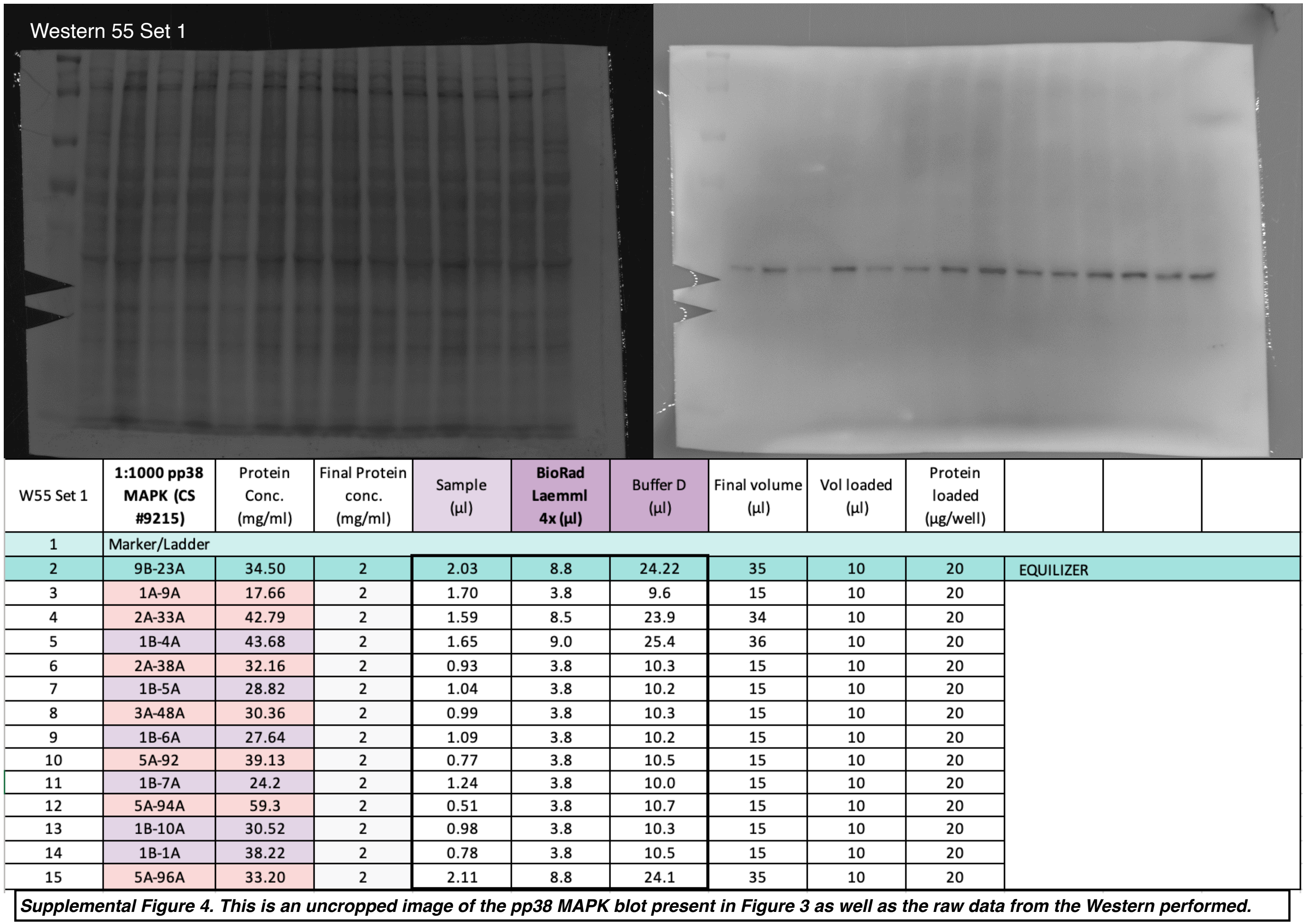

W55 Set 1	**1:1000 pp38 MAPK (CS #9215)**	Protein Conc. (mg/ml)	Final Protein conc. (mg/ml)	Sample (µl)	**BioRad Laemml 4x (µl)**	Buffer D (µl)	Final volume (µl)	Vol loaded (µl)	Protein loaded (µg/well)			
1	Marker/Ladder											
2	9B-23A	34.50	2	2.03	8.8	24.22	35	10	20	EQUILIZER		
3	1A-9A	17.66	2	1.70	3.8	9.6	15	10	20			
4	2A-33A	42.79	2	1.59	8.5	23.9	34	10	20			
5	1B-4A	43.68	2	1.65	9.0	25.4	36	10	20			
6	2A-38A	32.16	2	0.93	3.8	10.3	15	10	20			
7	1B-5A	28.82	2	1.04	3.8	10.2	15	10	20			
8	3A-48A	30.36	2	0.99	3.8	10.3	15	10	20			
9	1B-6A	27.64	2	1.09	3.8	10.2	15	10	20			
10	5A-92	39.13	2	0.77	3.8	10.5	15	10	20			
11	1B-7A	24.2	2	1.24	3.8	10.0	15	10	20			
12	5A-94A	59.3	2	0.51	3.8	10.7	15	10	20			
13	1B-10A	30.52	2	0.98	3.8	10.3	15	10	20			
14	1B-1A	38.22	2	0.78	3.8	10.5	15	10	20			
15	5A-96A	33.20	2	2.11	8.8	24.1	35	10	20			

Supplemental Figure 4. This is an uncropped image of the pp38 MAPK blot present in Figure 3 as well as the raw data from the Western performed.

Supplementary Figure 5

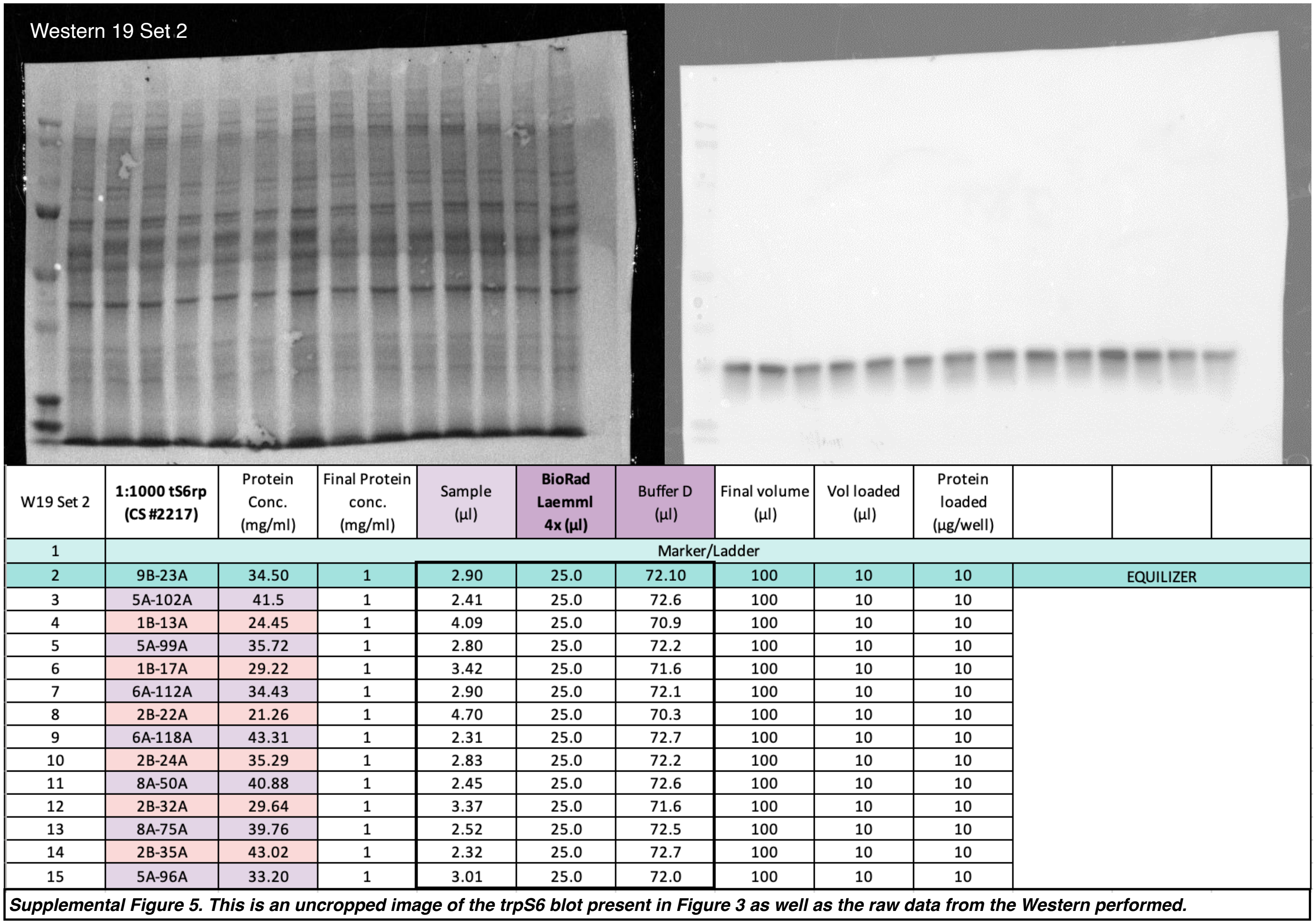

W19 Set 2	1:1000 tS6rp (CS #2217)	Protein Conc. (mg/ml)	Final Protein conc. (mg/ml)	Sample (µl)	BioRad Laemml 4x (µl)	Buffer D (µl)	Final volume (µl)	Vol loaded (µl)	Protein loaded (µg/well)			
1	Marker/Ladder											
2	9B-23A	34.50	1	2.90	25.0	72.10	100	10	10		EQUILIZER	
3	5A-102A	41.5	1	2.41	25.0	72.6	100	10	10			
4	1B-13A	24.45	1	4.09	25.0	70.9	100	10	10			
5	5A-99A	35.72	1	2.80	25.0	72.2	100	10	10			
6	1B-17A	29.22	1	3.42	25.0	71.6	100	10	10			
7	6A-112A	34.43	1	2.90	25.0	72.1	100	10	10			
8	2B-22A	21.26	1	4.70	25.0	70.3	100	10	10			
9	6A-118A	43.31	1	2.31	25.0	72.7	100	10	10			
10	2B-24A	35.29	1	2.83	25.0	72.2	100	10	10			
11	8A-50A	40.88	1	2.45	25.0	72.6	100	10	10			
12	2B-32A	29.64	1	3.37	25.0	71.6	100	10	10			
13	8A-75A	39.76	1	2.52	25.0	72.5	100	10	10			
14	2B-35A	43.02	1	2.32	25.0	72.7	100	10	10			
15	5A-96A	33.20	1	3.01	25.0	72.0	100	10	10			

Supplemental Figure 5. This is an uncropped image of the trpS6 blot present in Figure 3 as well as the raw data from the Western performed.

Supplementary Figure 6

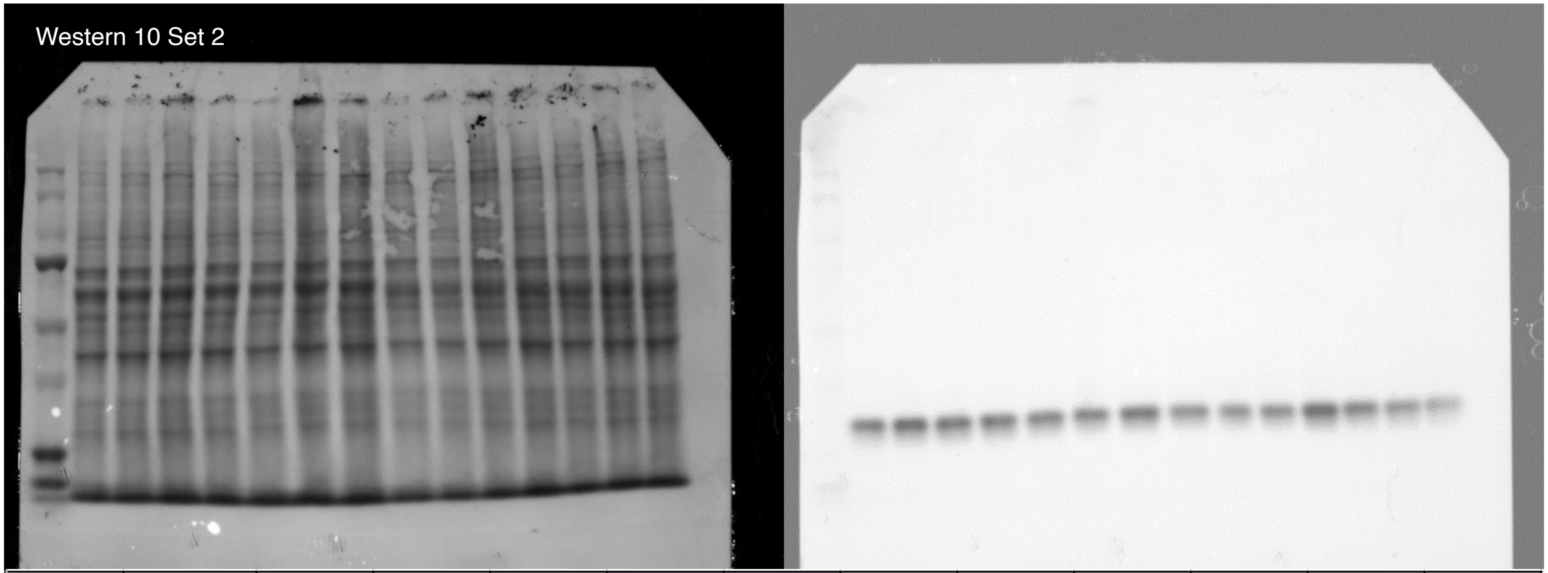

W10 Set 2	**1:1000 prpS6 (CS #2211)**	Protein Conc. (mg/ml)	Final Protein conc. (mg/ml)	Sample (µl)	**BioRad Laemml 4x (µl)**	Buffer D (µl)	Final volume (µl)	Vol loaded (µl)	Protein loaded (µg/well)			
1	Marker/Ladder											
2	9B-23A	34.50	1	2.90	25.0	72.10	100	10	10	EQUILIZER		
3	5A-102A	41.5	1	2.41	25.0	72.6	100	10	10			
4	1B-13A	24.45	1	4.09	25.0	70.9	100	10	10			
5	5A-99A	35.72	1	2.80	25.0	72.2	100	10	10			
6	1B-17A	29.22	1	3.42	25.0	71.6	100	10	10			
7	6A-112A	34.43	1	2.90	25.0	72.1	100	10	10			
8	2B-22A	21.26	1	4.70	25.0	70.3	100	10	10			
9	6A-118A	43.31	1	2.31	25.0	72.7	100	10	10			
10	2B-24A	35.29	1	2.83	25.0	72.2	100	10	10			
11	8A-50A	40.88	1	2.45	25.0	72.6	100	10	10			
12	2B-32A	29.64	1	3.37	25.0	71.6	100	10	10			
13	8A-75A	39.76	1	2.52	25.0	72.5	100	10	10			
14	2B-35A	43.02	1	2.32	25.0	72.7	100	10	10			
15	8A-59A	37.04	1	2.70	25.0	72.3	100	10	10			

Supplemental Figure 6. This is an uncropped image of the prpS6 blot present in Figure 3 as well as the raw data from the Western performed.

Supplementary Figure 7

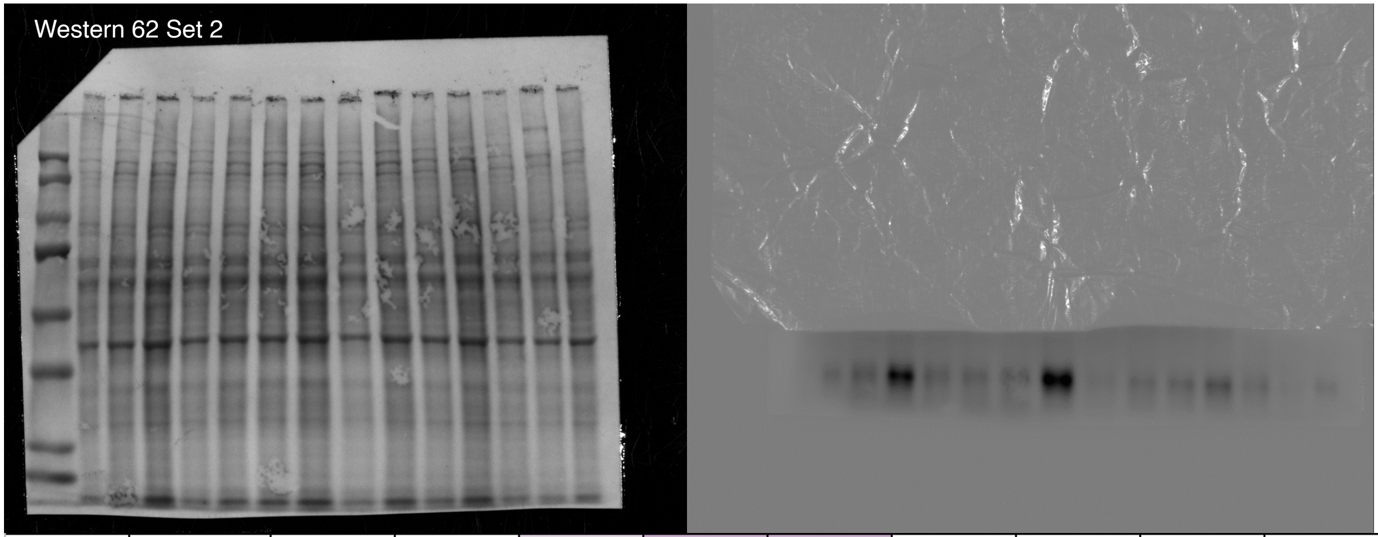

W62 Set 2	1:1000 t4EBP-1 (CS #9452)	Protein Conc. (mg/ml)	Final Protein conc. (mg/ml)	Sample (µl)	BioRad Laemml 4x (µl)	Buffer D (µl)	Final volume (µl)	Vol loaded (µl)	Protein loaded (µg/well)			
1	Marker/Ladder											
2	9B-23A	34.50	1	2.03	17.5	50.47	70	10	10	EQUILIZER		
3	5A-102A	41.5	1	0.72	7.5	21.8	30	10	10			
4	1B-13A	24.45	1	1.23	7.5	21.3	30	10	10			
5	5A-99A	35.72	1	0.84	7.5	21.7	30	10	10			
6	1B-17A	29.22	1	1.03	7.5	21.5	30	10	10			
7	6A-112A	34.43	1	0.87	7.5	21.6	30	10	10			
8	2B-22A	21.26	1	1.41	7.5	21.1	30	10	10			
9	6A-118A		1	0.69	7.5	21.8	30	10	10			
10	2B-24A	35.29	1	0.85	7.5	21.6	30	10	10			
11	8A-50A	40.88	1	0.73	7.5	21.8	30	10	10			
12	2B-32A	29.64	1	1.01	7.5	21.5	30	10	10			
13	8A-75A	39.76	1	0.75	7.5	21.7	30	10	10			
14	2B-35	43.02	1	0.70	7.5	21.8	30	10	10			
15	5A-96A	33.20	1	2.11	17.5	50.4	70	10	10			

Supplemental Figure 7. This is an uncropped image of the t4EBP-1 blot present in Figure 3 as well as the raw data from the Western performed.

Supplementary Figure 8

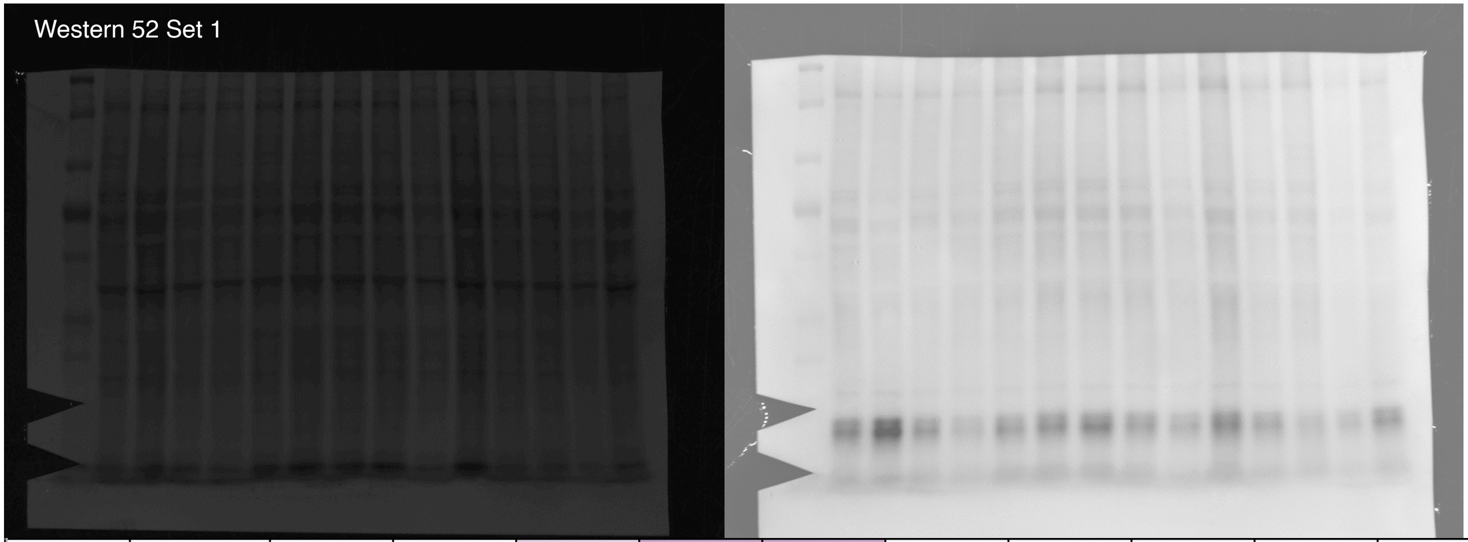

W52 Set 1	1:1000 p4EBP-1 (CS #9459)	Protein Conc. (mg/ml)	Final Protein conc. (mg/ml)	Sample (µl)	BioRad Laemml 4x (µl)	Buffer D (µl)	Final volume (µl)	Vol loaded (µl)	Protein loaded (µg/well)			
1	Marker/Ladder											
2	9B-23A	34.50	2	2.03	8.8	24.22	35	10	20	EQUILIZER		
3	1A-9A	17.66	2	1.70	3.8	9.6	15	10	20			
4	1B-1A	38.22	2	0.78	3.8	10.5	15	10	20			
5	2A-33A	42.79	2	0.70	3.8	10.5	15	10	20			
6	1B-4A	43.68	2	0.69	3.8	10.6	15	10	20			
7	2A-38A	32.16	2	0.93	3.8	10.3	15	10	20			
8	1B-5A	28.82	2	1.04	3.8	10.2	15	10	20			
9	3A-48A	30.36	2	0.99	3.8	10.3	15	10	20			
10	1B-6A	27.64	2	1.09	3.8	10.2	15	10	20			
11	5A-92	39.13	2	0.77	3.8	10.5	15	10	20			
12	1B-7A	24.2	2	1.24	3.8	10.0	15	10	20			
13	5A-94A	59.3	2	0.51	3.8	10.7	15	10	20			
14	1B-10A	30.52	2	0.98	3.8	10.3	15	10	20			
15	5A-96A	33.20	2	2.11	8.8	24.1	35	10	20			

Supplemental Figure 8. This is an uncropped image of the pEIF2a blot present in Figure 3 as well as the raw data from the Western performed.

Supplementary Figure 9

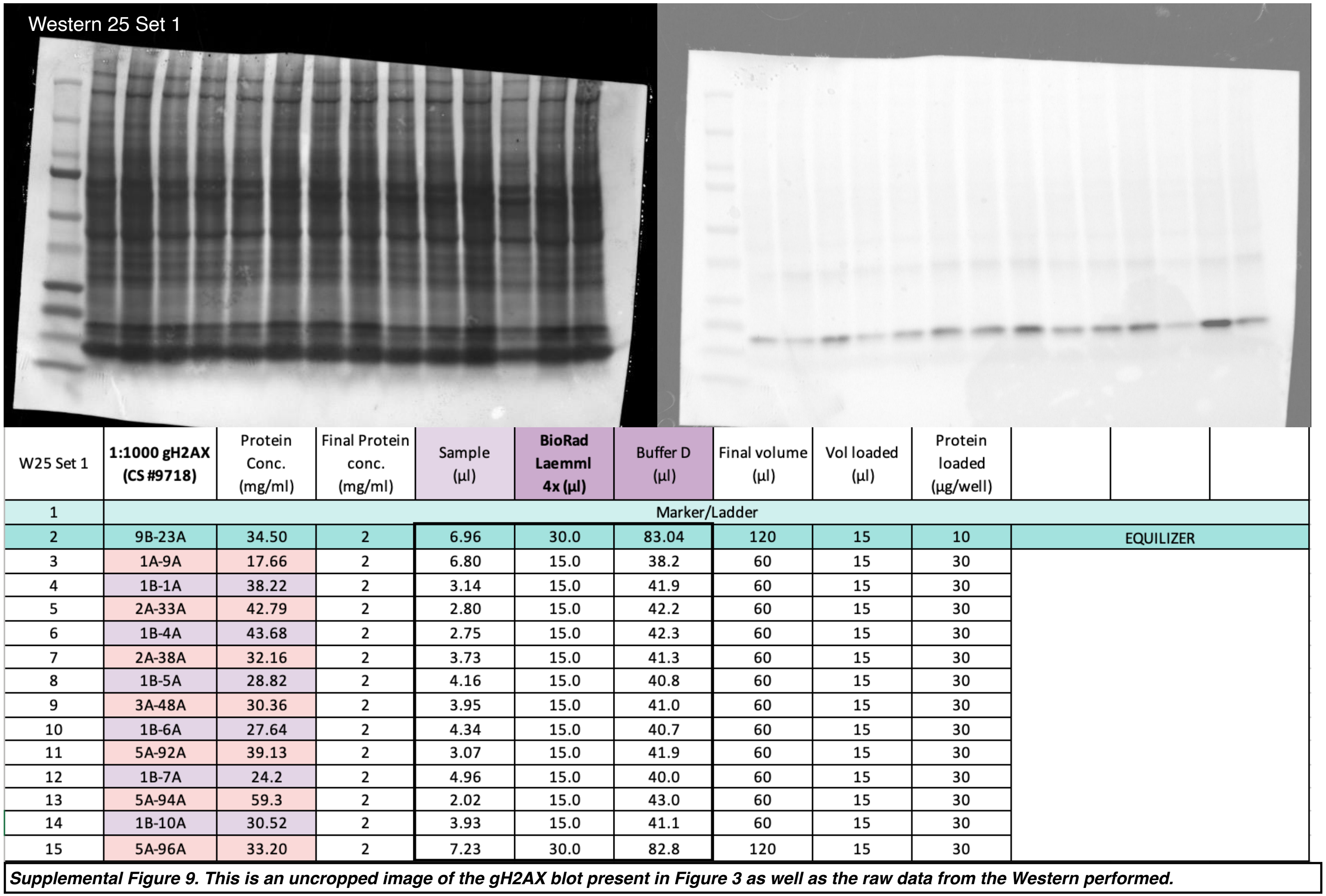

W25 Set 1	**1:1000 gH2AX (CS #9718)**	Protein Conc. (mg/ml)	Final Protein conc. (mg/ml)	Sample (µl)	**BioRad Laemml 4x (µl)**	Buffer D (µl)	Final volume (µl)	Vol loaded (µl)	Protein loaded (µg/well)			
1	Marker/Ladder											
2	9B-23A	34.50	2	6.96	30.0	83.04	120	15	10	EQUILIZER		
3	1A-9A	17.66	2	6.80	15.0	38.2	60	15	30			
4	1B-1A	38.22	2	3.14	15.0	41.9	60	15	30			
5	2A-33A	42.79	2	2.80	15.0	42.2	60	15	30			
6	1B-4A	43.68	2	2.75	15.0	42.3	60	15	30			
7	2A-38A	32.16	2	3.73	15.0	41.3	60	15	30			
8	1B-5A	28.82	2	4.16	15.0	40.8	60	15	30			
9	3A-48A	30.36	2	3.95	15.0	41.0	60	15	30			
10	1B-6A	27.64	2	4.34	15.0	40.7	60	15	30			
11	5A-92A	39.13	2	3.07	15.0	41.9	60	15	30			
12	1B-7A	24.2	2	4.96	15.0	40.0	60	15	30			
13	5A-94A	59.3	2	2.02	15.0	43.0	60	15	30			
14	1B-10A	30.52	2	3.93	15.0	41.1	60	15	30			
15	5A-96A	33.20	2	7.23	30.0	82.8	120	15	30			

Supplemental Figure 9. This is an uncropped image of the gH2AX blot present in Figure 3 as well as the raw data from the Western performed.
